# Discovery of a Novel Respiratory Syncytial Virus Replication Inhibitor

**DOI:** 10.1128/AAC.02576-20

**Published:** 2021-05-18

**Authors:** Li Wang, Qihui Zhu, Kunlun Xiang, Yaling Zhang, Baocun Li, Xin Yu, Guang Yang, Chungen Liang, Hongying Yun, Meifang Zhang, Ning Qin, Lu Gao

**Affiliations:** a Infectious Disease Discovery, Roche Pharmaceutical Research and Early Development, Roche Innovation Center Shanghai, Shanghai, China; b Medicinal Chemistry, Roche Pharmaceutical Research and Early Development, Roche Innovation Center Shanghai, Shanghai, China; c Lead Discovery, Roche Pharmaceutical Research and Early Development, Roche Innovation Center Shanghai, Shanghai, China

**Keywords:** respiratory syncytial virus, replication, inhibitor

## Abstract

A high-throughput screen of a Roche internal chemical library based on inhibition of the respiratory syncytial virus (RSV)-induced cytopathic effect (CPE) on HEp-2 cells was performed to identify RSV inhibitors. Over 2,000 hits were identified and confirmed to be efficacious against RSV infection *in vitro*. Here, we report the discovery of a triazole-oxadiazole derivative, designated triazole-1, as an RSV replication inhibitor, and we characterize its mechanism of action. Triazole-1 inhibited the replication of both RSV A and B subtypes with 50% inhibitory concentration (IC_50_) values of approximately 1 μM, but it was not effective against other viruses, including influenza virus A, human enterovirus 71 (EV71), and vaccinia virus. Triazole-1 was shown to inhibit RSV replication when added at up to 8 h after viral entry, suggesting that it inhibits RSV after viral entry. In a minigenome reporter assay in which RSV transcription regulatory sequences flanking a luciferase gene were cotransfected with RSV N/P/L/M2-1 genes into HEp-2 cells, triazole-1 demonstrated specific and dose-dependent RSV transcription inhibitory effects. Consistent with these findings, deep sequencing of the genomes of triazole-1-resistant mutants revealed a single point mutation (A to G) at nucleotide 13546 of the RSV genome, leading to a T-to-A change at amino acid position 1684 of the L protein, which is the RSV RNA polymerase for both viral transcription and replication. The effect of triazole-1 on minigenome transcription, which was mediated by the L protein containing the T1684A mutation, was significantly reduced, suggesting that the T1684A mutation alone conferred viral resistance to triazole-1.

## INTRODUCTION

Human respiratory syncytial virus (RSV) is an enveloped negative-sense single-stranded RNA virus that belongs to the Orthopneumovirus genus and Pneumoviridae family and is the major causative agent of severe lower respiratory tract infection in infants and children, especially those with chronic lung disease or congenital heart disease (1, 2). RSV also induces potentially life-threatening pulmonary disease in elderly and immunocompromised adults (3). RSV infection accounts for an estimated 160,000 deaths worldwide every year (4). Therefore, preventive and therapeutic strategies to treat RSV infection are urgently needed.

Efforts to develop an RSV vaccine to prevent this disease were initiated in the 1960s, but there is currently no safe and effective vaccine available due to the negative track record of RSV vaccine-enhanced disease, early age of infection, virus-induced immune evasion, and lack of an *in vivo* model supporting natural infection (5). Palivizumab (Synagis), a humanized monoclonal antibody specific to the RSV fusion (F) protein that blocks viral entry, was approved as the prophylaxis agent (6). However, it did not prevent infection of the lower respiratory system in allogeneic hematopoietic stem cell transplantation patients (7), and its high cost has limited its widespread use. Ribavirin has been used as an anti-RSV treatment. A review of the data from available clinical trials showed that ribavirin may reduce mechanical ventilation duration and hospitalization time, but the patient number in these clinical trials was small, and the quality of the clinical data was questioned (8). In the last decade, significant progress has been made in identification of novel RSV inhibitors, and most studies have focused on inhibitors that target the RSV F protein to block viral entry. Several very potent fusion inhibitors have been discovered. These include the biphenyl analogues CL387626 and RFI-641 (9–11), the bis-tetrazole-benzhydrylphenol derivatives VP-14637 and MDT637 (12), the azabenzimidazole BMS-433771 (13, 14), the benzimidazole derivative TMC-353121 (15), imidazoisoindolone BTA-9881 (U.S. patent application no. 12/443,177), and GS-5806 (16, 17). Development of most of these molecules has been discontinued for different reasons (16–18). The development of GS-5806 (presatovir) was recently terminated because the treatment did not improve virological or clinical outcomes versus placebo in hematopoietic cell transplant (HCT) patients with RSV lower respiratory tract infection (LRTI) (19).

Although blocking viral entry and cell-to-cell spread could inhibit the spread of the virus to uninfected cells, an entry inhibitor has limited effects on cells that are already inoculated. This limitation could be addressed by targeting viral replication. A functional RSV RNA replication complex consists of the following four viral proteins: the nucleocapsid protein (N), the large protein (L), the phosphoprotein (P), and matrix protein 2-1 (M2-1). The N protein binds to genomic and antigenomic RNA to form helical nucleocapsids, which serve as the templates for RNA synthesis; the L protein is an RNA-dependent RNA polymerase that is responsible for synthesis of viral RNAs and mRNA capping; the P protein interacts with the N, L, and M2-1 proteins and facilitates the replication complex formation; and the M2-1 protein is a transcription antitermination factor (20–25). Inhibitors of the N and L proteins have been identified. RSV604 is a small-molecule inhibitor targeting the N protein, and it inhibits both viral RNA synthesis and infectivity of the released virus (26, 27). Adult hematopoietic stem cell transplant patients who had drug exposure exceeding the *in vitro* 90% effective concentration (EC_90_) had more substantial viral load reductions and symptom improvement; however, the drug’s pharmacokinetic properties in humans are poor, and no further reports were released (28). EDP-938 is a potent replication inhibitor that is currently in phase II clinical trial. It may also target the N protein but is >20-fold more potent than RSV604 (29). ALN-RSV01 is a small interfering RNA (siRNA) that targets RSV N protein mRNA and that was also advanced to phase II trials; unfortunately, no statistically significant reduction in viral load was observed in lung transplant patients with confirmed RSV infection (30). The other confirmed druggable target is the L protein. Given these important roles, inhibition of L protein function may lead to efficient suppression of viral replication. Moreover, the L protein is highly conserved across the nonsegmented negative-sense RNA virus family (31). These traits make the L protein an excellent target. The nucleoside analog ALS-8176, which targets the RSV polymerase specifically but does not inhibit human RNA polymerase, showed excellent efficacy in humans in a phase IIa RSV challenge study (32). These findings demonstrated the efficacy of an RSV replication inhibitor in infected patients for the first time. However, the development of ALS-8176 was later discontinued due to unrevealed reasons. Two other nucleoside analogs were reported, but they are still in the early preclinical stage (33, 34). Nonnucleot(s)ide drugs are another type of L polymerase inhibitor. An *in vitro* polyadenylation-dependent capture assay was developed with the crude RNP (RNA polymerase complex) from RSV-infected HEp-2 cells to screen for L polymerase transcription inhibitors (35). A nonnucleot(s)ide transcriptase inhibitor, designated compound A, was identified with this assay (36). YM-53403, another RSV L inhibitor, was identified through a HeLa cell-based cytopathic effect (CPE) assay screen. This compound targets L polymerase, as shown by the identification of a single point mutation in the L polymerase (Y1631H) in a YM-53403-resistant virus (37). Three compounds that share a similar chemical scaffold with YM-53403 were later reported. AZ-27 showed significantly improved potency (38). Compound 4a demonstrated *in vivo* efficacy in a cotton rat infection model (39). Pulmocide (PC786) was developed as an inhaled therapy and advanced to phase II trial in hematopoietic stem cell transplant patients (40, 41); however, the phase II TreatRSV1 trial was later discontinued due to patient recruitment issues that could not be resolved. Discovery of novel L polymerase inhibitors of different chemical structures are urgently needed.

In this study, to identify novel small-molecule RSV replication inhibitors, we performed high-throughput screening (HTS) with a Roche internal library using the CPE assay on HEp-2 cells and discovered a novel chemical scaffold small-molecular inhibitor, Triazole-1, which can suppress RSV replication at single-digit micromolar 50% inhibitory concentrations (IC_50_). The time-dependent drug addition experiment and the DNA sequence analysis of triazole-1-resistant mutants suggested that this compound targets a unique region in RSV L polymerase. Triazole-1 is a highly promising chemical scaffold for developing a novel anti-RSV drug.

## RESULTS

### Identification of RSV inhibitors through HTS.

**FIG 1:**
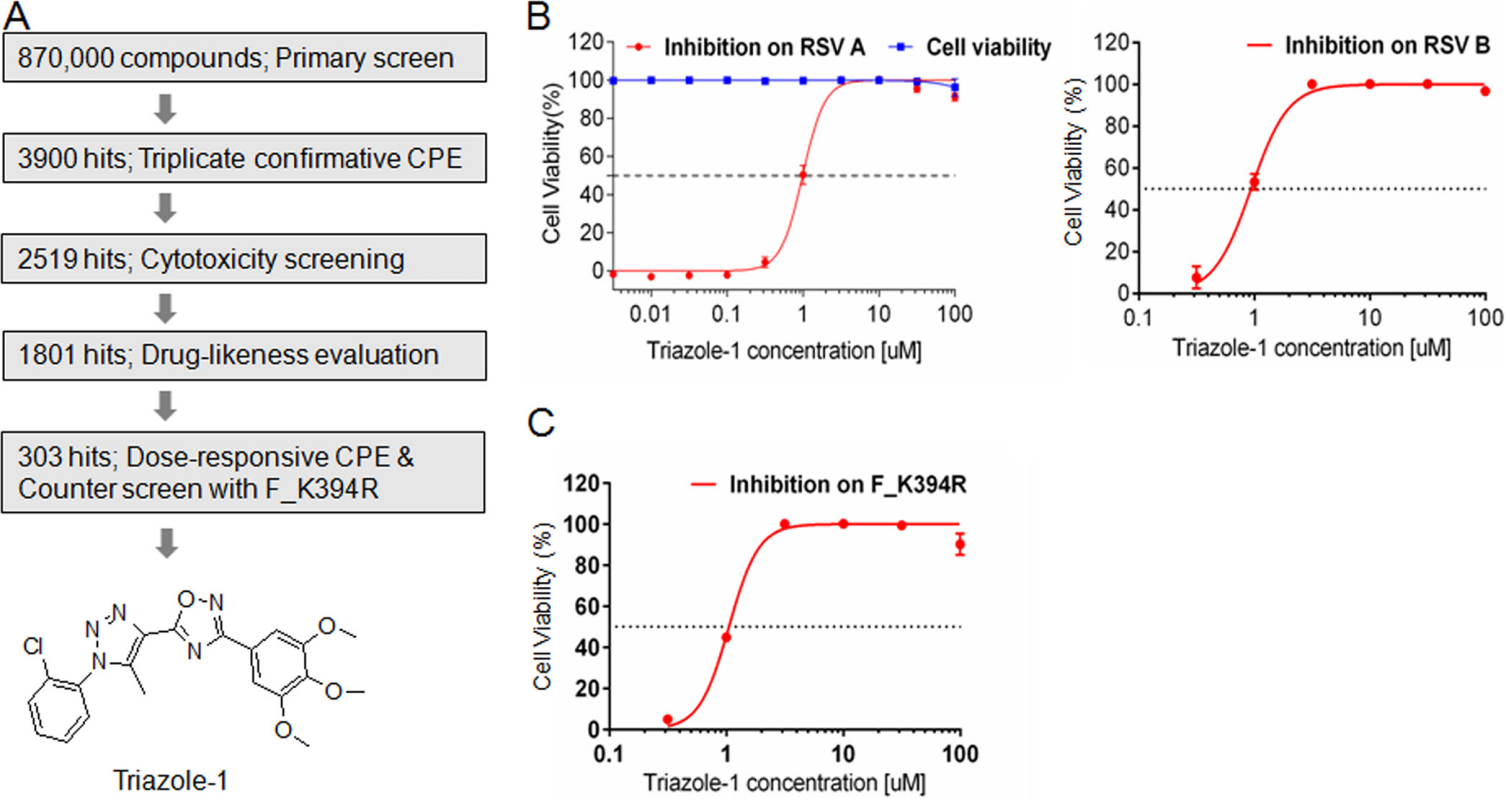
Discovery of triazole-1 as an RSV inhibitor from high-throughput screening. (A) The screen cascade for identifying RSV replication inhibitors using CPE assay-based high-throughput screening and the structure of Triazole-1. (B) Triazole-1 inhibition of RSV subtypes A and B in CPE assays and the effect of triazole-1 on cell viability. (C) Triazole-1 inhibition of the F_K394R variant virus in the CPE assay. Anti-RSV activity of the compounds is expressed as a percentage of viable cells compared with dimethyl sulfoxide (DMSO)-treated cells Each data point represents mean ± standard deviation from three replicates. The experiments were repeated in three independent experiments.

To identify novel small-molecular inhibitor scaffolds that specifically target RSV replication, we designed an HTS based on the RSV CPE assay. In this assay, the compounds were added 2 h after addition of the virus to increase the possibility of identifying postentry inhibitors. A Roche library of approximately 870,000 compounds was screened at a single concentration of 10 μM in this assay (Fig. 1A). Approximately 3,900 hits were identified from the primary screening when the cutoff was set at >40% inhibition compared with the dimethyl sulfoxide (DMSO) control. Among these, 2,519 hits showed reproducible anti-RSV activities in the triplicate confirmation assay (65% confirmation rate). The effect of these hits on cell viability was evaluated at 10 μM, 1,801 hits that showed <20% cytotoxicity were subjected to medicinal chemistry drug-likeness evaluation, and 303 hits were chosen for the subsequent evaluation in the dose-responsive CPE assay to determine the IC_50_ and 50% cell cytotoxicity (CC_50_) values. Because the K394R mutation in the fusion protein confers resistance to at least two fusion inhibitors with different structures, BMS-433771 and TMC353121 (13, 42), the CPE assay with this mutant virus (designated F_K394R) was used as a counterscreen assay to potentially filter out some entry inhibitors. A total of 58 hits that showed similar inhibition against F_K394R virus and wild-type virus were subjected to further characterization. Among them, one hit, designated triazole-1, showed an approximate IC_50_ of 1.0 μM on both RSV A and B subtypes, while it did not show cytotoxicity at concentrations up to 100 μM (Fig. 1B). Triazole-1 showed a similar IC_50_ (1.1 μM) in the CPE assay with the F_K394R mutant virus (Fig. 1C), indicating it might be a postentry inhibitor. This compound was subjected to further mechanism of action characterization.

### Triazole-1 inhibits RSV at a postentry step.

**FIG 2:**
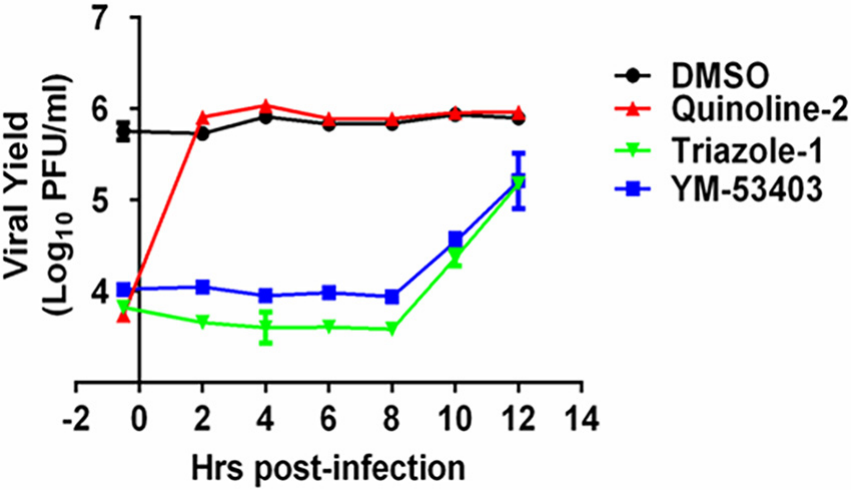
Time of addition experiment of triazole-1. HEp-2 cells were infected with RSV (multiplicity of infection [MOI] = 5). Triazole-1, YM53403, and quinoline-2 were added at the indicated times. At 24 h postinfection, viral yield in cell culture supernatant was determined by plaque assays and expressed as log_10_ PFU/ml. Each data point represents mean ± standard deviation from three replicates. The experiments were repeated in two independent experiments.

To gain a better understanding of the mechanism of action of triazole-1, we performed a time-dependent drug addition experiment. In this experiment, the cells were infected at a relatively high multiplicity of infection (MOI) of 5. Each compound was added at a concentration of approximately 10-fold over its IC_90_ to the cells to ensure that maximal inhibition could be achieved at different time points postinoculation. We found that 20 μM triazole-1 inhibited viral replication at similar levels when it was added to the cells at 0.5 h prior to virus inoculation or up to 8 h after the infection (Fig. 2). YM-53403, the previously identified RSV L polymerase inhibitor, was used as the positive control and showed similar results to those shown by triazole-1. As expected, a Roche fusion inhibitor (43), designated quinoline-2, showed a significant reduction in viral titer, but it lost inhibitory activity when it was added at 2 h postinfection or later time points. These data strongly suggest that the anti-RSV activity of triazole-1 occurs in a step after viral penetration, and it may inhibit the transcription and/or replication of the RSV genome.

### Triazole-1 inhibits N/P/L/M2-1-mediated RSV transcription.

**FIG 3:**
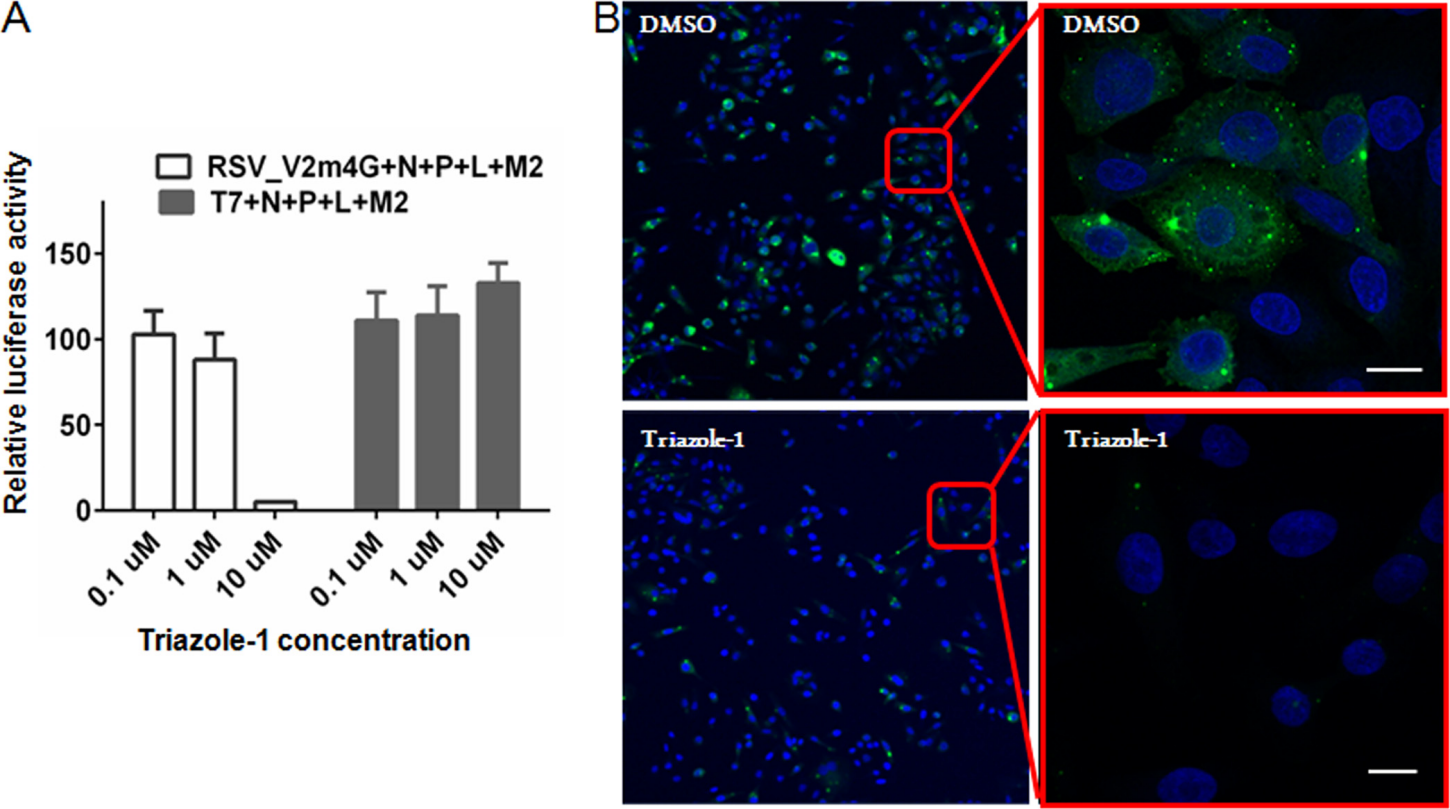
Triazole-1 inhibits RSV replication. (A) The effect of triazole-1 on RSV N/P/L/M2-1 protein-driven RSV minigenome transcription. Luciferase assays were performed on HEp-2 cells, which were transfected with a minigenome (Minigenome-Luc) construct or T7 promoter construct (T7pro-Luc) together with pcDNA constructs expressing N, P, L, and M2-1 proteins, and then treated with DMSO or triazole-1 (0.1 μM, 1 μM, and 10 μM). The graph shows the percentage of luciferase activity relative to DMSO (DMSO is set as 100%). Each data point represents mean ± standard deviation from three replicates. The experiments were repeated in two independent experiments. (B) Confocal microscopy of RSV-infected HEp-2 cells that were treated with DMSO or 10 μM triazole-1 and stained for the RSV replication complex (green) and nuclei (blue). The top panel shows the RSV replication foci in DMSO-treated cells, and the bottom panel shows the RSV replication foci in triazole-1-treated cells. Bars, 10 μM.

Next, an N/P/L/M2-1-driven minigenome luciferase reporter assay was established based on a previously published similar assay (44) and was used to investigate if triazole-1 could inhibit RSV transcription mediated by the four-protein transcriptase complex. Triazole-1 specifically decreased the N/P/L/M2-1-mediated RSV promoter activation, while had no effect on a T7 promoter-controlled luciferase expression at up to 10 μM (Fig. 3A), suggesting that triazole-1 blocks N/P/L/M2-1-mediated RSV promoter transcription events but does not inhibit cellular transcription events. The effect of triazole-1 on RSV replication foci was also investigated by immunofluorescence. HEp-2 cells were inoculated with RSV at an MOI of 0.5 and left for 16 h to allow the initial establishment of replication foci. Then, 10 μM triazole-1 was added to the cell monolayer. At 24 h posttreatment, the number of replication foci was significantly reduced (Fig. 3B), further confirming that this compound suppresses RSV replication.

### The antiviral effect of triazole-1 on other viruses.

**FIG 4:**
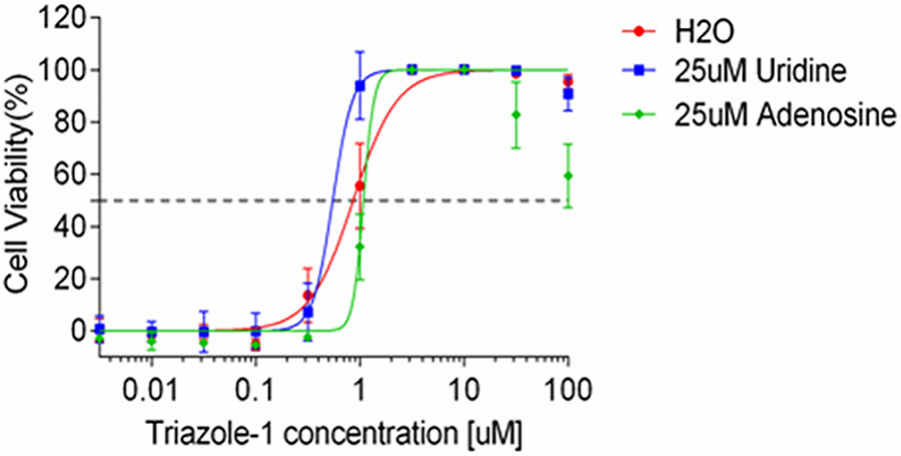
The effect of purine or pyrimidine on anti-RSV activity of triazole-1. Anti-RSV activity of triazole-1 was evaluated in RSV CPE assays in the presence of 25 μM uridine or adenosine. Anti-RSV activity of the compounds is expressed as a percentage of viable cells compared with DMSO-treated cells. Each data point represents mean ± standard deviation from three replicates. The experiments were repeated in two independent experiments.

To evaluate the specificity of the anti-RSV activity by triazole-1, we evaluated triazole-1 inhibition of a panel of different viruses, including influenza virus A, human enterovirus 71 (EV71), and vaccinia virus, using CPE assays. No significant inhibition was found for these viruses (Table 1). Since triazole-1 shares certain chemical structure similarities with an isoxazolepyrazole series, which was reported to inhibit a broad spectrum of viruses that included RSV by targeting cellular dehydrogenases and blocking the *de novo* pyrimidine biosynthesis pathway (45), a purine/pyrimidine rescue experiment was performed to test whether the mechanism of action of triazole-1 is the same as that of the isoxazolepyrazole series. Excess amounts of uridine or adenosine (25 μM) were added together with triazole-1 in the CPE assay. Both had no effect on the anti-RSV activity of triazole-1 (Fig. 4) and cell viability (data not shown), demonstrating that triazole-1 inhibits RSV replication via a mechanism that is independent of pyrimidine biosynthesis.

**TABLE 1:** Effects of triazole-1 on influenza virus A, human enterovirus 71, and vaccinia virus in the CPE assay

Virus (strain)	Cells	EC_50_ (μM)	CC_50_ (μM)
Influenza (A/Weiss/43)	MDCK	>100	>100
EV71 (Shenzhen/120F1/09)*^a^*	RD	>100	>100
Vaccinia (VP13)	HFF	>100	>100

a EV, enterovirus.

### Identification of the viral target of triazole-1.

**FIG 5:**
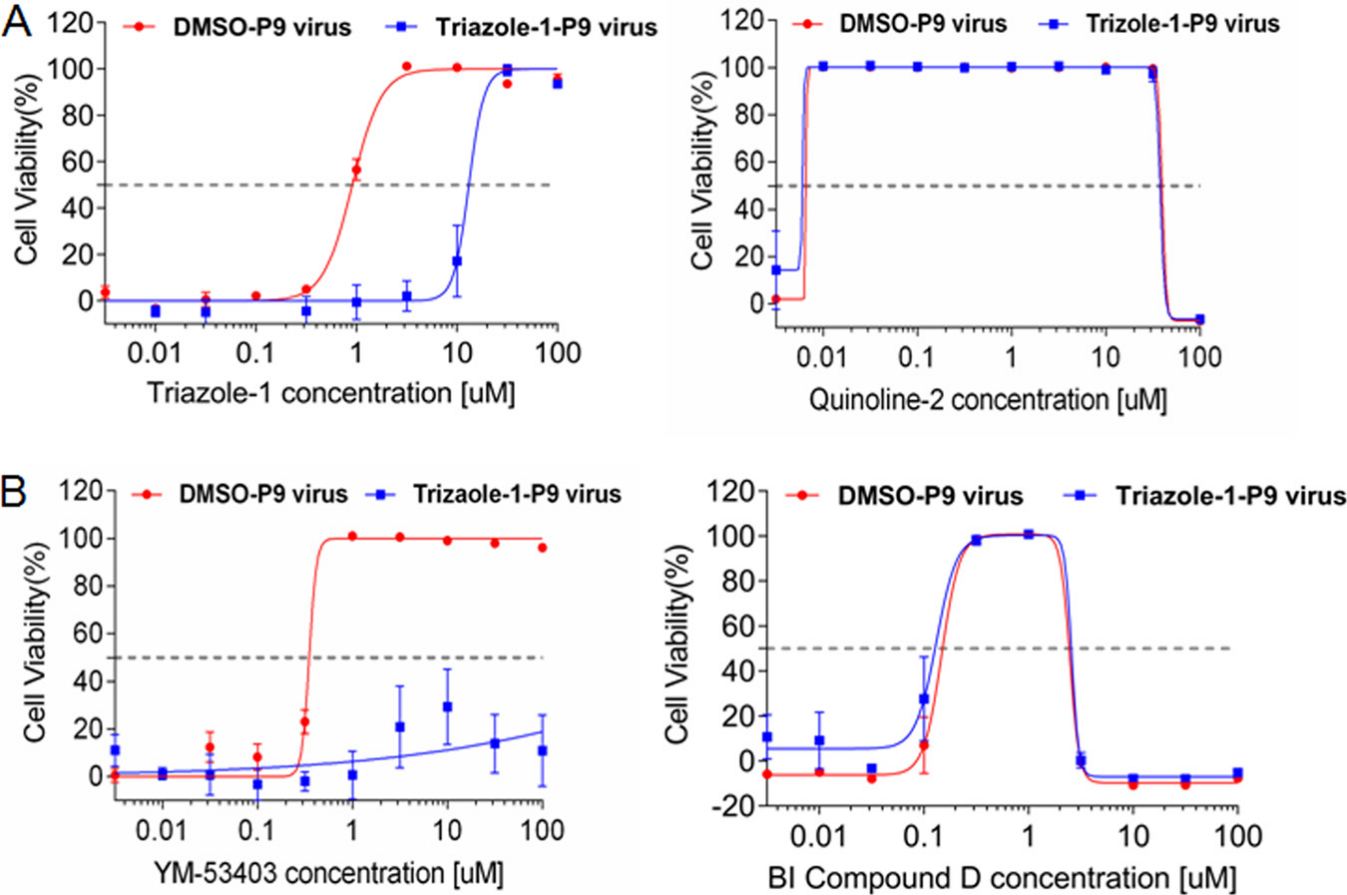
Identification of the T1684A mutation on L polymerase in the triazole-1-resistant variant. (A) Triazole-1 and quinoline-2 inhibition of the DMSO-p9 and Triazole-1-p9 viruses. (B) YM-53403 and BI compound D inhibition of the DMSO-p9 and Triazole-1-p9 viruses. Anti-RSV activity of the compounds is expressed as percentage of viable cells compared with DMSO-treated cells. Each data point represents mean ± standard deviation from three replicates. The experiments were repeated in three independent experiments.

To potentially identify the viral molecular target of triazole-1, we cultured the virus in the presence of increasing concentrations of the compound to generate drug-resistant mutants. When all cells died from infection, the virus was passaged to an uninfected cell monolayer. After 9 passages, an ∼21-fold increase in the IC_50_ of triazole-1 was observed (the virus was designated triazole-1-p9); however, the IC_50_ of triazole-1 did not shift for the DMSO-selected virus (designated DMSO-p9) under similar culture conditions (Fig. 5A). As expected, the fusion inhibitor control quinoline-2 displayed similar inhibitory effects on both the triazole-1-p9 and DMSO-p9 viruses, further confirming that triazole-1 has a different mechanism from that of the fusion inhibitor. To determine whether a mutation in a specific amino acid is responsible for the resistance, we plaque purified the triazole-1-p9 virus four times, and three independent clones were subjected to both whole-RSV-genome Sanger sequencing and deep sequencing with a 454 GS Junior sequencer (Roche). One single mutation at nucleotide 13546 (GenBank accession no. AY911262), which leads to a T-to-A change at amino acid 1684 of L polymerase, was found in all three clones but not in DMSO-p9 virus clones (see Fig. S1 in the supplemental material). The effect of YM-53403 and another previously reported replication inhibitor from Boehringer Ingelheim (designated BI compound D) on triazole-1-p9 was tested. YM-53403 completely lost its inhibitory effects, while the inhibitory activity of BI compound D against triazole-1-p9 was preserved (Fig. 5B). Together, these data suggest that triazole-1 specifically targets RSV L polymerase and may target different regions from that targeted by BI compound D. The resistance of YM-53403 to triazole-1-resistant virus indicates the T1684A mutation had an impact on the YM-53403 binding pocket on L polymerase.

**FIG 6:**
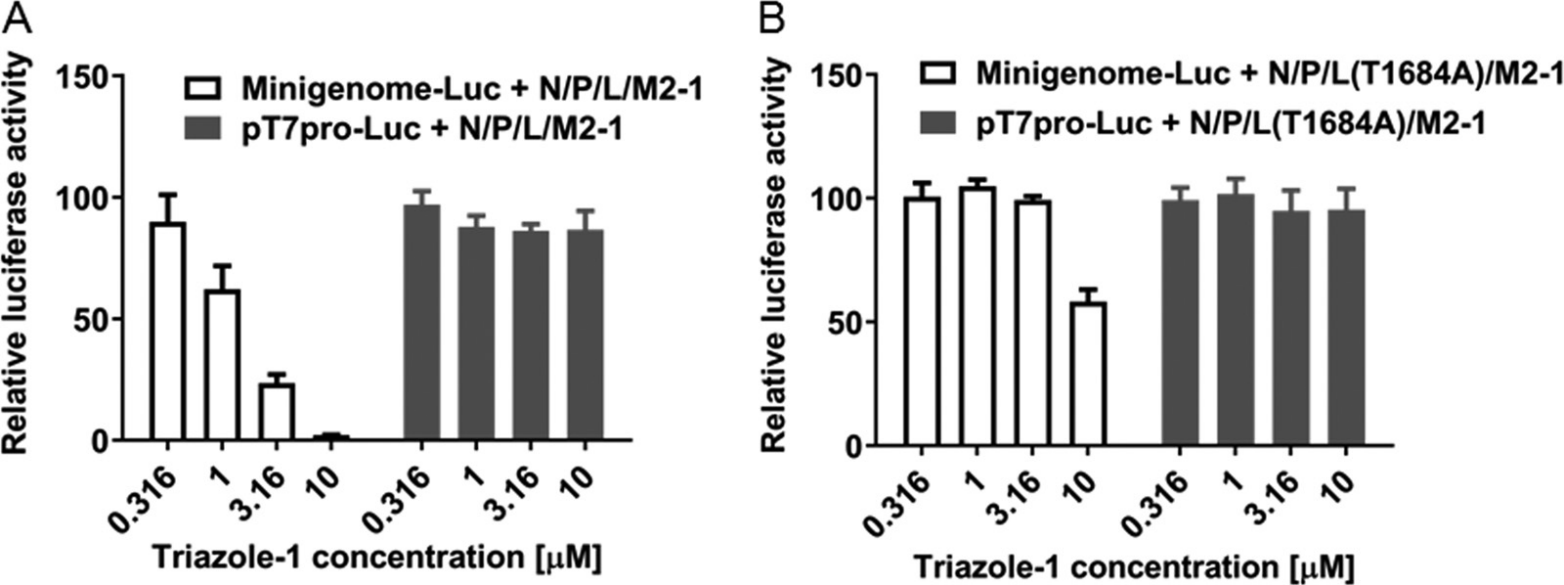
Effect of triazole-1 on RSV minigenome transcription mediated by wild-type L protein or the T1684A mutant. Luciferase assays were performed on HEp-2 cells transfected with a minigenome construct (Minigenome-Luc) or T7 promoter construct (T7pro-Luc) together with pcDNA constructs expressing N, P, L (wild-type or T1684A mutant), and M2-1 proteins and treated with 0.316 μM to 10 μM triazole-1. The graph shows the relative luciferase activity compared with DMSO. Each data point represents mean ± standard deviation from three replicates. The experiments were repeated in three independent experiments.

To further confirm if the T1684A mutation in the triazole-1-p9 virus alone contributed to the resistance to triazole-1, we introduced the mutation into the L expression construct, and the inhibitory effects of triazole-1 on the mutant protein L(T1684A)-driven minigenome expression were tested. L(T1684A) showed similar activation of RSV minigenome transcription as the wild-type L protein (data not shown). However, the inhibitory effect of triazole-1 on the mutant protein-driven minigenome expression was significantly reduced (Fig. 6). These data confirm that the T1684 residue of the RSV L polymerase is critical for the RSV replication inhibition by triazole-1.

## DISCUSSION

In the last decade, pharmaceutical companies and research laboratories have attempted to discover and develop new RSV treatments due to an important unmet medical need. The promising data for the RSV fusion inhibitor GS-5806 and the polymerase inhibitor ALS-8176 in a healthy volunteer challenge study in phase II studies have demonstrated the concept of using small-molecule drugs to relieve RSV-caused symptoms (16, 17); however, both assets were later terminated for different reasons. Compared with entry inhibitors, replication inhibitors may have an advantage in terms of treatment window, as demonstrated by *in vitro* experiments by different laboratories (37, 38, 41, 46) and in our study (Fig. 2), as well as by clinical data from the nucleoside analog ALS-8176. ALS-8176 inhibits RSV polymerase activity by inducing RNA synthesis chain termination (47) and showed superior virological outcomes compared to those of the RSV fusion inhibitor GS-5806 (16, 32). Nucleoside analogs are known to be associated with toxicities, such as peripheral neuropathy, myopathy, pancreatitis, and lactic acidosis with hepatic steatosis, primarily due to mitochondrial and nuclear genotoxicity (48). Nonnucleoside RNA polymerase inhibitors may not have these concerns, as they directly target the virus-specific polymerase.

Thus far, only a few nonnucleoside RSV L polymerase inhibitors have been discovered (35, 37, 39, 49). One major obstacle to identification of L polymerase inhibitors is the lack of an easy and straightforward assay system. Biochemical assays with crude RNA polymerase lysate have been established for screening transcriptase inhibitors (35), but other functions, such as replicase activity, could not be determined in this assay. The replicon assay is robust in identifying replication inhibitors (38), but construction of replicons and establishment of replicon cell lines are needed. Another approach to identifying L polymerase inhibitors is a phenotypic screen with an RSV infection assay. The advantage of this approach is that it is in the real virus replication setting. However, it is associated with the challenge of differentiating postentry inhibition from entry inhibition at the screening step. In our study, we employed a novel counterscreen assay in which inhibition of the F_K394R mutant virus replication by compounds was tested to filter out a part of the fusion inhibitors. The rationale for using the F_K394R mutant virus is that the mutation was reported to confer resistance to different chemical types of fusion inhibitors (14, 42). This approach has significantly expedited the identification of nonfusion inhibitors. To the best of our knowledge, this is the first report using this method as a filter to facilitate the identification of RSV replication inhibitors.

Previously, three different chemical scaffolds of nonnucleoside L polymerase inhibitors, represented by BI compound D, YM-53403, AZ-27, compound 4a, and AVG-233 have been discovered (35, 37–40, 49, 50). Triazole-1 is the fourth novel chemical scaffold L inhibitor reported thus far. Resistance virus selection and characterization demonstrated that this compound targets a different amino acid residue (L_T1684) from the other classes of L polymerase inhibitors. Triazole-1 most likely has a different mechanism of action from that of BI compound D, which targets cotranscriptional mRNA guanylylation (36), as T1684 is distant from the L protein mutations in compound D-resistant viruses (I1381S, E1269D, and L1421F). This hypothesis is in line with our data showing that compound D displayed similar inhibitory effects on triazole-1-resistant viruses as those on the wild-type virus (Fig. 5A). In contrast, YM-53403 showed no inhibition of the triazole-1-resistant virus (Fig. 5B), suggesting that mutation of the T1684 residue may induce change(s) in the binding pocket of YM-53403. It is likely that AZ-27 and compound 4a are expected to show a similar loss of inhibitory effects on triazole-1-resistant viruses because AZ-27 and YM-53403 share a similar chemical scaffold and genetic resistance profile (38). The molecular mechanism of AZ-27 was recently reported to be inhibition of the transcriptional initiation of both mRNA and genomic RNA (51). Whether triazole-1 also inhibits transcription or initiation or other transcription steps requires further investigation. Since T1684 is very close to the methyltransferase domain, it is reasonable to hypothesize that the anti-RSV effect of triazole-1 is mediated through the inhibition of mRNA capping. In the future, studies should be performed to assess this hypothesis. Since the cryo-EM structures of RSV L polymerase in complex with the P phosphoprotein has been solved (52), it might be worth to exploring if triazole-1 could bind to RSV L/P complex and if the complex structure could be solved.

Notably, triazole-1 inhibits both subtype A (long strain) and subtype B virus (18537 strain) (Fig. 1C). This result is consistent with the identification of L(T1684A) as the resistant mutation for triazole-1, as T1684A and its neighboring sequences are highly conserved in subtype A and subtype B viruses. More importantly, L(T1684A) variants were not found in the RSV sequences currently deposited in GenBank, suggesting that the likelihood of naturally occurring resistance against triazole-1 is low. Of course, concerns over the potential of rapid generation of resistance mutants in clinical applications due to the rapid appearance of resistant L(T1684A) variants in the *in vitro* experiment are reasonable (see Fig. S1 in the supplemental material). This issue is associated with RSV inhibitors of essentially all mechanism of actions, as genetic drift is an intrinsic characteristic of RNA viruses. One important question to be answered in the future is that of the fitness of the L(T1684A) mutant compared with the wild-type virus. Meanwhile, a feasible approach to increase the resistance barrier is combination therapy. For example, one class of replication inhibitor can be combined with another class of replication inhibitor or with a fusion inhibitor. This concept has been demonstrated *in vitro* with the combination of the nucleoside inhibitor ALS-8112 and the nonnucleoside polymerase inhibitor AZ-27, which showed significant synergistic inhibition on RSV replication (53).

The functions of the RSV L polymerase have not been fully elucidated. The reasons include limited understanding of the replication complex structure and difficulty in reconstituting an *in vitro* assay with purified L and other replication proteins. Small-molecule inhibitors have been shown to be useful for identifying novel motifs and characterizing the function of L protein subdomains (36). Triazole-1 also offers a good tool to further elucidate the structure and function of the L protein. Moreover, in our study, the minigenome assay was successfully used to verify if the T1684A mutation alone is sufficient to cause resistance to triazole-1, demonstrating that the minigenome assay is a very useful and convenient reverse genetics tool.

In summary, we identified a novel chemical scaffold that could inhibit the replication of RSV subtype A and B viruses with submicromolar IC_50_ values by targeting the L polymerase. Triazole-1 provides a good starting point for further optimization and generation of improved compounds. In addition, triazole-1 targets a novel region of the L polymerase and could serve as a useful tool to deepen the understanding of RSV L polymerase structure and functions.

## MATERIALS AND METHODS

### Cells.

HEp-2 cells were acquired from the American Type Culture Collection (ATCC) and maintained in Dulbecco’s minimal essential medium (DMEM)/F12 medium supplemented with 10% fetal bovine serum (FBS) and 1% penicillin-streptomycin. MDCK cells were obtained from the ATCC and cultured in Eagle’s minimal essential medium with 10% FBS and 1% penicillin-streptomycin. HFF cells were obtained from the ATCC and cultured in DMEM with 10% FBS and 1% penicillin-streptomycin. RD cells were purchased from the cell bank of CAS-TCHU 45.

### Viruses.

RSV subtype A virus (long strain) and subtype B virus (18537 strain) and influenza virus (A/Weiss/43 strain) were obtained from the ATCC. Human EV71 (Shenzhen/120F1/09 strain) was a gift from Tianwei Lin (Xiamen University, China). Vaccinia virus (VP13 strain) was obtained from the China Center for Type Culture Collection (CCTCC). F_K394R mutant viruses were plaque purified from RSV long strain viruses after multiple passages in the presence of an RSV fusion inhibitor (unpublished data). The mutation was confirmed by sequencing.

### Plasmids.

The minigenome reporter plasmid with luciferase as the reporter gene (Minigenome-Luc) was constructed as described previously (44). The T7pro-Luc control plasmid was constructed by TOPO cloning of the full-length luciferase gene into pCR2.1 (Invitrogen). Luciferase DNA was amplified using pGL3 (Promega) as a template with the following two primers: Luc2P-F, 5′-GACTGCCATGGTTAGACGTTGATCCTGGCGC-3′, and Luc2P-R, 5′-GACTGCATATGATGGAAGATGCCAAAAACATTAA-3′.

The expression plasmids for the N, P, L, and M2-1 proteins were constructed by cloning the full-length coding sequence into pcDNA3.1(+). N DNA was amplified by PCR using RSV long strain cDNA with the forward primer (N-F) 5′-GACTAGCTAGCATGGCTCTTAGCAAAGTCAAG-3′ containing an NheI site and the reverse primer (N-R) 5′-GACTGGATCCTCAAAGCTCTACATCATTATCT-3′ containing a BamHI site (underlining indicates the respective restriction sites). The PCR product was digested with NheI and BamHI and ligated into NheI/BamHI-digested pcDNA3.1(+) to generate pcDNA-N. pcDNA-P, pcDNA-L, and pcDNA-M2-1 were constructed similarly with the following primers: P-F (containing an MluI site), 5′-GACTGACGCGTATGGAAAAGTTTGCTCCTGAA-3′; P-R (containing an XhoI site), 5′-GACTGCTCGAGTCAGAAATCTTCAAGTGATAG-3′; L-F (containing an MluI site), 5′-GACTGACGCGTATGGATCCCATTATTAATGGA-3′; L-R (containing an XhoI site), 5′-GACTGCTCGAGTTATTCATTATGAAAGTTGTATAA-3′; M2-1-F (containing a KpnI site), 5′-GACTAGGTACCATGTCACGAAGGAATCCTTG-3′; and M2-1-R (containing a BamHI site), 5′-GACTGGATCCTCAGGTAGTATCATTATTTTTG-3′. L(T1684A) mutant plasmid was constructed with a QuikChange II XL site-directed mutagenesis kit (Agilent) using the following primers: T1684A-F, 5′-CTTCTCAGATAATGCTCATCTATTAAC-3′, and T1684A-R, 5′-GTTAATAGATGAGCATTATCTGAGAAG-3′.

### Compounds.

Triazole-1, triazole-2, quinoline-2, YM-53404, and BI compound D were synthesized in-house.

### High-throughput screening.

HEp-2 cells were seeded on 384-well plates at a density of 1,500 cells in 30 μl culture medium per well using Bravo (Agilent) and incubated overnight at 37°C and 5% CO_2_. Appropriate amounts of viruses (∼1× 90% tissue culture infective dose [TCID_90_]) in 15 μl culture medium were added to each well to produce over 90% CPE after 4 days of incubation. Then, 5 μl of diluted compound was added in culture medium by Bravo. After 4 days of incubation, cell viability was assessed using the CCK-8 kit (Dojindo). A virus control (no compound addition) and a cell control (no virus or compound addition) were included to provide the minimum value and the maximum value, respectively, for calculating the percentage of inhibition. For analysis of compound cytotoxicity, cells were incubated with compounds in the absence of virus. Anti-RSV activity of the compounds is expressed as a percentage of viable cells compared with those exposed to DMSO.

### RSV dose-response CPE assay.

HEp-2 cells were seeded on 96-well plates at a density of 6,000 cells per well and incubated overnight at 37°C and 5% CO_2_. Serial dilutions of compounds made with DMSO were further diluted with culture medium before they were added to the cells to reach the desired final compound concentrations and 1% DMSO concentration. Appropriate amounts of viruses (∼1× TCID_90_) were added to each well for 24 h to produce over 90% CPE after 5 days. At 5 days postinfection, cell viability was assessed using a CCK-8 kit (Donjindo). The virus control and cell control were the same as those described above. Anti-RSV activity and cytotoxicity of the compounds were evaluated in the same way as described above. The IC_50_ or CC_50_ values were derived from the dose-response curve using Prism software.

### CPE assay of influenza, EV71, and vaccinia viruses.

Assay procedures were similar to those for the RSV CPE assay except for the cell types, cell seeding number, inoculum, days of compound treatment, and cell viability detection method. These data are listed in Table S1 in the supplemental material.

### Time of addition.

HEp-2 cells were seeded in 48-well plates (5 × 10^4^ cells/well) and incubated overnight at 37°C and 5% CO_2_. RSV (long strain) was added to the cell monolayer at an MOI of 5 and incubated for 2 h at 37°C. Cells were then washed three times to remove virus anchoring on the cell surface. Compounds were added to the cells either 0.5 h before viral inoculation or at different time points after viral inoculation. DMSO-treated cells served as the negative control. Cell supernatants were harvested at 24 h after infection and subjected to viral titer determination by plaque assays.

### Minigenome assay.

HEp-2 cells seeded on 6-well plates were transfected with the minigenome reporter construct Minigenome-Luc and plasmids expressing N, P, L, and M2-1 using Lipofectamine 2000 (Invitrogen) according to the transfection manual. T7pro-Luc was used for assessing the cytotoxicity control. The ratio of the plasmid amount was 2:2:2:1:1 Minigenome-Luc or T7pro-Luc:pcDNA-N:pcDNA-P:pcDNA-L:pcDNA-M2-1. At 4 h posttransfection, cells from each well were mixed with 0.5 × 10^4^ PFU of vTF7 and resuspended in 2.2 ml medium. The cell suspension was seeded in 96-well plates (63 μl/well). Compound dilutions were then made and added (DMSO concentration in the medium, 1%). After 48 h of compound treatment, cells were subjected to luciferase assays using the Bright-Glo luciferase assay system (Promega). The percentage of inhibition was normalized to DMSO control samples.

### Immunofluorescence.

Cells seeded on 4-well chamber slides were infected with RSV at the MOI of 0.1 for 24 h, fixed with 4% paraformaldehyde for 15 min, and permeabilized with 0.5% Triton X-100 for 10 min. Cells were incubated with RSV3 antibody (1:200 dilution; Novocastra) in blocking buffer (Tris-buffered saline with Tween 20 [TBST] with 5% bovine serum albumin [BSA]) at room temperature for 1 h, followed by incubation with Alexa Fluor 488 donkey anti-mouse immunoglobulin (Invitrogen) for 30 min. Cell nuclei were stained with 2 mg/ml Hoechst 33342 for 10 min after secondary antibody incubation. All images were obtained with a LSM710 Zeiss confocal microscope. Images were taken in a fixed setting with 20× (for overview) and 63× objectives (for zoomed-in images).

### Drug-resistant mutant selection.

Triazole-1-resistant viruses were isolated by culturing RSV (long strain) in HEp-2 cells in the presence of increasing concentrations of triazole-1 (from 2 μM to 24 μM). When the entire cell monolayer was infected, an appropriate amount of supernatant was transferred to an uninfected cell monolayer in a T25 flask. The DMSO-treated virus was generated by culturing the virus with the same concentration of DMSO (1% dimethyl sulfide [DMS]) to provide a genetic drift control. The susceptibility of these viruses to triazole-1 was examined by CPE assays.

### Deep sequencing.

Viral RNA was extracted from RSV virus stocks using a QIAamp viral RNA kit (Qiagen), and cDNA was then synthesized by Moloney murine leukemia virus (M-MuLV) reverse transcriptase (NEB), using hexamer as a primer. A total of 25 overlapping PCR products (∼400 to 600 bp in length) covering the full-length RSV genome were amplified using the cDNA as a template with the primers shown in Table S2 in the supplemental material. After purification by a QIAquick PCR purification kit (Qiagen) and quantification by Quant-iT PicoGreen double-stranded DNA (dsDNA) reagent and kits (Invitrogen), the 25 PCR products were mixed in a 1:1 ratio to achieve a total amount of no less than 1 μg and a concentration of ∼50 ng/μl. The DNA was then subjected to deep sequencing by a 454 GS Junior sequencer (Roche Diagnostics) according to the designated manual.

### Data availability.

The newly determined sequences have been deposited in GenBank under accession no. MW768127 and MW768128.
