# Supplementary material for: Discovery of a Novel Respiratory Syncytial Virus Replication Inhibitor
Source: Antimicrob Agents Chemother. 2021 May 18;65(6):e02576-20. doi: 10.1128/AAC.02576-20 (PMC8316115; doi:10.1128/AAC.02576-20)
Supplement: Supplementary file 1 [file aac.02576-20-s0001.pdf]

| **Virus** | **Virus inoculum** | **Cell type** | **Cell seeding number** | **Compound treatment time** | **Cell viability detection** |
|---|---|---|---|---|---|
| Influenza | 0.008 MOI | MDCK | 6000/well | 3 days | MTT |
| EV71 | 0.12 MOI | RD | 8,000/well | 3 days | CCK-8 |
| Vaccinia | 0.05 MOI | HFF | 25,000/well | 4 days | CCK-8 |

Table S1. Information for CPE assay of influenza, EV71, and vaccinia viruses.

| Primer | Sequence location (5' to 3') | Start | End |
|---|---|---|---|
| SeqF1-F | ACGCGAAAAAATGCGTACAACAA | 1 | 23 |
| SeqF1-R | TCTCAAGTGACAACGGTCTCATGTC | 676 | 700 |
| SeqF2-F | GGACACAACCCACAATGATACCAC | 630 | 653 |
| SeqF2-R | CCCAGTGAATTTATGATTAGCATCT | 1304 | 1328 |
| SeqF3-F | CCTAATTATGATGTGCAGAAACACA | 1245 | 1269 |
| SeqF3-R | AAGACTCCCCACCGTAGCATTA | 1906 | 1927 |
| SeqF4-F | TTGAAGGGATTTTTGCAGGATTG | 1855 | 1877 |
| SeqF4-R | GTTCCCTGCATTATCATCTGTCTCA | 2546 | 2570 |
| SeqF5-F | AACCAAAGAAAGCCCTATAACATCA | 2495 | 2519 |
| SeqF5-R | TTTTGGATTGGCTGGTTGTTTT | 3180 | 3201 |
| SeqF6-F | CCCACTCATCCAACCAAACATC | 3131 | 3152 |
| SeqF6-R | GATGGCATTTTTGAATTCAGTGG | 3805 | 3827 |
| SeqF7-F | CCAACATACCTAAGATCCATCAGTG | 3741 | 3765 |
| SeqF7-R | GTTTGTTTAGTATTGCAGTCATGATG | 4406 | 4431 |
| SeqF8-F | GCAAATTCTGGCCTTACTTTACACT | 4336 | 4360 |
| SeqF8-R | TCCTGGTGTTGTTGAAGCTAGTATG | 5025 | 5049 |
| SeqF9-F | CAGCTTGGAATCAGCTTCTCCA | 4972 | 4993 |
| SeqF9-R | TTGCATTTGCTTTGAGGATTGG | 5669 | 5690 |
| SeqF10-F | CACAAAAGGCCATGACCAACTC | 5603 | 5624 |
| SeqF10-R | TTCTGCAGCTTTGCTTATTCACA | 6277 | 6299 |
| SeqF11-F | CCAGCAAAGTGTTAGACCTCAAAAA | 6225 | 6249 |
| SeqF11-R | TTTGCCATAGCATGACACAATGG | 6894 | 6916 |
| SeqF12-F | AACAGATGTAAGCAGCTCCGTTATC | 6856 | 6880 |
| SeqF12-R | GTTAATCTGGTATTCAATTGTGTTTTA | 7545 | 7571 |
| SeqF13-F | AAAATCTGAACTTCATCGAAACTCTT | 7464 | 7489 |
| SeqF13-R | TGGCATGGTCATTTGTATCACTAAC | 8145 | 8169 |
| SeqF14-F | TCCACAAGAGCATAACCATCAACA | 8104 | 8127 |
| SeqF14-R | TAGCAATCTGTTCCAACGAGGTC | 8787 | 8809 |
| SeqF15-F | AAAGGTGAAATAAAATTAGAAGAGCCT | 8722 | 8748 |
| SeqF15-R | TGAATCCGCATCTTAAGCCTAAG | 9390 | 9412 |
| SeqF16-F | CATGGATTAGTAACTGCTTGAACACA | 9353 | 9378 |
| SeqF16-R | CGATAGAAACGTAGTCCTGATAACAC | 10039 | 10064 |
| SeqF17-F | TCCTTCTTTGTTGGAACTTACAGAAA | 10002 | 10027 |
| SeqF17-R | GGAAAATAGAGATTGTACACCATGCA | 10664 | 10689 |
| SeqF18-F | GCATTTCGATATGAAACGTCATGT | 10615 | 10638 |
| SeqF18-R | CGCATGATTTTTTAATTGTAGAGCA | 11307 | 11331 |
| SeqF19-F | GAGGTGAAAGTCTATTATGCAGTTTAA | 11249 | 11275 |
| SeqF19-R | GCAGAAGTCTTTTCCAGTATGTTAGT | 11938 | 11963 |

| | | | |
|---|---|---|---|
| SeqF20-F | AAGTTTACCCTTTTATAAAGCAGAGAA | 11877 | 11903 |
| SeqF20-R | AAGCTGGTATTGATGCAGGGAAT | 12540 | 12562 |
| SeqF21-F | ATTTGCATCGCCTTACAGTCAGTA | 12503 | 12526 |
| SeqF21-R | TGAAGTGTTCATATCACACTCCAGTT | 13154 | 13179 |
| SeqF22-F | TCTTGTGTTTTCATAAAGGTTATGGC | 13121 | 13146 |
| SeqF22-R | TCACAACCGTAGGGAATAGATTGTA | 13798 | 13822 |
| SeqF23-F | GAACCAATTACAGCAAACAAGACCT | 13772 | 13796 |
| SeqF23-R | TTGCATTTTCTTACATGCTTGCTC | 14352 | 14375 |
| SeqF24-F | TGATGCCGAATTGCCTGTAACA | 14301 | 14322 |
| SeqF24-R | CATGATTGAACCACTTTAAGATGTTCA | 14807 | 14833 |
| SeqF25-F | GCTGGACGGAATGAAGTTTTCAG | 14758 | 14780 |
| SeqF25-R | ACGAGAAAAAAAGTGTCAAAAACTAAT | 15200 | 15226 |

Table S2. Information of the primers for deep sequencing.

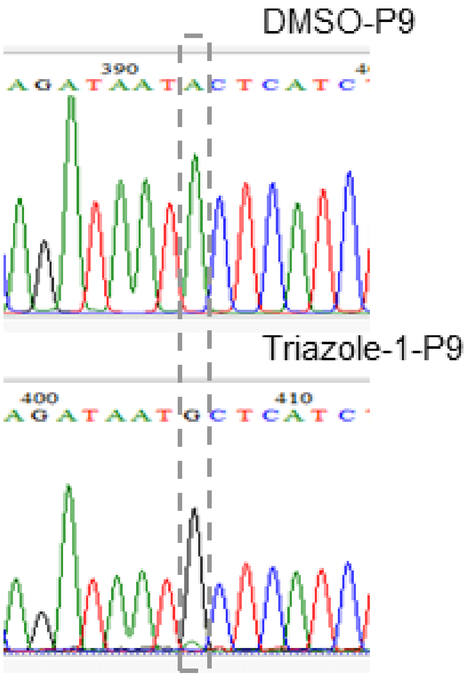


Fig. S1. Mutation of A to G at nucleotide 13546 in the RSV genome (GenBank accession No: AY911262)
